# Influence of Adhesive in FSW: Investigation on Fatigue Behavior of Welded, Weld-Bonded, and Adhesive-Bonded Joints in Aluminum AA 6082 T6

**DOI:** 10.3390/ma12081242

**Published:** 2019-04-16

**Authors:** Enrico Lertora, Chiara Mandolfino, Marco Pizzorni, Carla Gambaro

**Affiliations:** Department of Mechanical Engineering, Polytechnic School, University of Genoa, Via All’ Opera Pia 15, 16145 Genoa, Italy; e.lertora@unige.it (E.L.); m.pizzorni@dime.unige.it (M.P.); gambaro@dime.unige.it (C.G.)

**Keywords:** weld-bonding, friction stir welding, adhesive bonding, lap-joints, quasi-static tests, dynamic tests

## Abstract

Friction stir welding (FSW) is a solid-state technique, which has assumed an increasingly important role in automotive, naval, and aeronautical industry over the years. Nowadays, thanks to its several benefits, FSW is used to weld any type of metallic, polymeric, or composite material. In recent decades, adhesive bonding has also enhanced relevance due to a request for much lighter structures to increase performance without increasing fuel consumption. From a mechanical perspective, welding has a high tensile strength despite a low fatigue resistance through the lack of joint elasticity. Therefore, the aim of this study is to investigate and compare static and dynamic behavior of welded, weld-bonded, and adhesive-bonded joints. After choosing the most suitable adhesive, surface preparation, consisting of sandblasting, was carried out. First of all, on the basis of previous experience in FSW, the process parameters of hybrid welding were determined. Both quasi-static and dynamic behavior of welded, adhesive-bonded, and weld-bonded joints, made in overlapped configuration, were then compared. Experimental tests showed that the adhesive limits the negative effect, due to the presence of the structural notch of FSW overlapped joints.

## 1. Introduction

Weld-bonding is a hybrid method of assembly that merges together both welding and adhesive bonding processes. The aim of this combination is essentially to maximize the advantages of both processes while reducing the disadvantages [1]. 

In particular, the friction stir welding process (FSW), developed at The Welding Institute (TWI), is a solid state joining process, which means that the materials are joined below melting point temperatures. This aspect brought about the extreme advantage of enabling the joining of previously non-weldable materials with good mechanical properties, low distortions, good surface finish, and high automation potential. FSW has been and still is a “hot topic” of studies concerning its applicability on high-strength materials, polymers and composites, the optimization of process parameters, and its comparison with other conventional techniques [2,3,4].

FSW is inherently suitable for the configuration of a butt joint; in fact, the motion of the tool is ideal for mixing the material arranged on two sides of the same plane. Problems arise for welding in lap-joint configuration, because, in this case, the sheets to be welded overlap and, therefore, the stirred material must be moved from top to bottom and no longer from right to left (or vice versa) [5]. In fact, the limited mixing zone in the plane separating the sheets causes the formation of a hook shape [6,7,8]. This defect is in fact a perfect trigger for the propagation of cracks and it could constitute a possible cause of localized corrosion phenomena. Furthermore, it affects the static strength of the welded beam and, in particular, strongly limits its resistance to fatigue stress.

Another joining technique, with a longer history in industrial application, is structural bonding. Adhesive bonding is particularly suitable for lightening-oriented applications, in performing sectors such as aerospace, motor racing, and nautical. Indeed, it presents many benefits compared to traditional joining technology: Strong reduction of distortions, especially in the junction of very thin and precision parts, possibility of sound heterogeneous joints, even using substrates that are completely different to one another, and making complicated geometries [9,10]. Furthermore, adhesive-bonded joints guarantee a homogeneous stress distribution and, thus, absence of strain concentrations within the adhesives. Unfortunately, relating to joints’ mechanical performance, they could provide some technological issues, like sensitivity to temperature and aging, that are not easy to manage. In recent years, structural bonding is an extremely active research field, and its studies are leading to important developments [11,12,13]. Nevertheless, it cannot be said that comparable reliability has been achieved to that of welded joints that guarantee metallic continuity [14].

Therefore, the use of a hybrid joining technology, which combines adhesive bonding and FSW, could overcome the problems presented by each of the two technologies and allow a designer to use a joint that is simultaneously resistant to dynamic loads but ensures a certain degree of safety from the point of view of aging sensitivity. This technology is commonly called weld-bonding, and presents several advantages, such as an improvement of fatigue strength, since it reduces the stress concentration in the notch. It also improves energy absorption, it reduces temperature and humidity sensitivity, and it can avoid localized corrosion phenomena. Furthermore, stresses in hybrid joints are lower and more uniform than for welded joints. This provides an increase of in-plane tensile shear and/or compressive buckling load [1].

At present, many results on the application of traditional fusion welding and resistance spot welding (RSW) and adhesives of various kinds for the realization of hybrid joints are reported [15,16,17,18,19,20,21,22,23,24]. On the contrary, from a general review of existing literature, a systematic experimental investigation on the difference of mechanical behavior between hybrid and FS-welded or adhesive-bonded joints considering both static and dynamic behavior continues to be lacking. Few works report the use of the same hybrid technologies, mainly due to Braga et al. [25,26] and Fortunato et al. [6]. Braga et al. focus on the development of the manufacturing process of hybrid FSW and AB aluminum lap joints. In particular, static strength and distortion levels of the manufactured joints were assessed and compared to FSW-only and AB-only joints. The results established that hybrid FSW and adhesive bonding produced higher strength and more ductile lap joints than FSW lap joints, although not as strong or ductile as adhesive-bonded joints. In another study, Fortunato et al. found the application of ultrasonic testing to friction stir weld-bonded joint inspection a successful method to evaluate joint quality. 

This work reports a thorough and completely innovative investigation of the comparison between two possible hybrid-joint production methods. In particular, mechanical behavior through quasi-static and fatigue tests was evaluated and compared with only welded and adhesive-bonded joints. This information, to the knowledge of the authors, is currently not available in the literature.

This work focused on hybrid joints, created by combining epoxy adhesive and FS-Welded joints, therefore getting friction stir weld-bonded joints. Results of the presence of the adhesive within the welded joint are evaluated through quasi-static and dynamic mechanical resistance. 

## 2. Materials and Methods 

The aim of this study is the investigation of the feasibility of friction stir weld-bonding of aluminum substrates. The material used as substrate is a 2 mm thick AA6082 alloy in T6 condition. Table 1 reports its chemical composition and Table 2 its mechanical properties. The sheet was cut and prepared in pieces 250 mm in length and 100 mm in width. Specific surface preparations for each type of joint were made and these are reported below. Specimens for macrographic investigation were collected from the transverse section of welded and weld-bonded joints. The specimens were mechanically polished with various grades of emery paper from coarse to fine. Then, polished specimens were etched by immerging them in Keller’s reagent (95 mL water, 2.5 mL HNO_3_, 1.5 mL HCl, 1.0 mL HF) for 120–150 s, followed by washing with a stream of water and then drying with hot air to reveal the structure of weld metal. Then the investigations were carried out under an optical microscope (Leica MZ6, Leica Microsystems, Milan, Italy).

**Figure 1:**
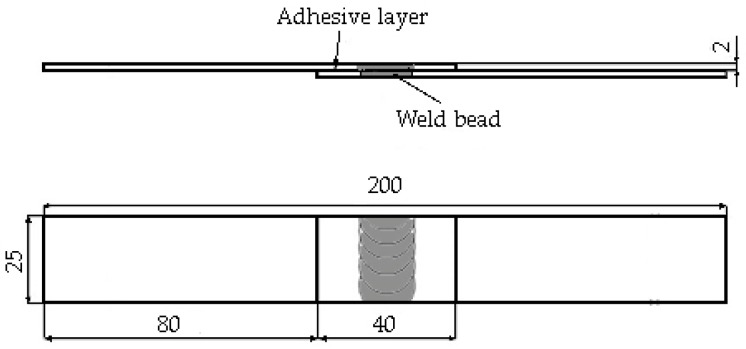
Configuration of a single overlapping friction stir weld (FSW)-bonded joint (values in mm).

Once made, the joints were cut into samples suitable for mechanical investigations. All kinds of samples were made using the same geometry, which is reported in Figure 1, with weld-bonded samples as an example.

Even if the related standard recommends an overlap length of 12.7 mm for adhesive-bonded joints, since both welded and adhesive-bonded joints must have the same geometry, it was necessary to increase the overlap area in order to have enough space to contain both welded seam and adhesive on the two sides. It was decided, based on the diameter of the shoulder of the available FSW tool, to have a 40 mm overlap length.

### 2.1. Adhesive Bonding (AB)

For all joints where adhesive is expected to be used (all the joints except for only welded ones), the overlap area is superficially treated with acetone in order to clean the surface of contaminants and sandblasted to increase surface roughness. By treating the surface, the adhesion between aluminum and adhesive is improved and, therefore, the sealing of the joint increases considerably [27]. Sandblasting was carried out with a Lampugnani model LC/S sandblasting machine (Lampugnani, Assago (MI), Italy). It was decided to use 120 mesh abrasive alumina particles so that the grain size of the abrasive was fine enough not to affect the surface too severely.

For the adhesive bonding (AB) joints, the adhesive was applied over the overlapping area of the joints, creating a thickness of 0.5 mm of adhesive, in order to be able to compare in the same conditions all types of joints requiring the presence of adhesive. To keep the gap between the plates constant, two sheets of 0.5 mm thickness were placed on the sides and pressure was applied along the entire adhesive-bonded length by means of a metal bar in order to spread the excess adhesive.

The adhesive used was a two-component epoxy resin (3M-DP490, Table 3). The complete crosslinking of the adhesive was achieved at room temperature in one week, as suggested by the manufacturer.

### 2.2. Friction Stir Welding (FSW)

**Figure 2:**
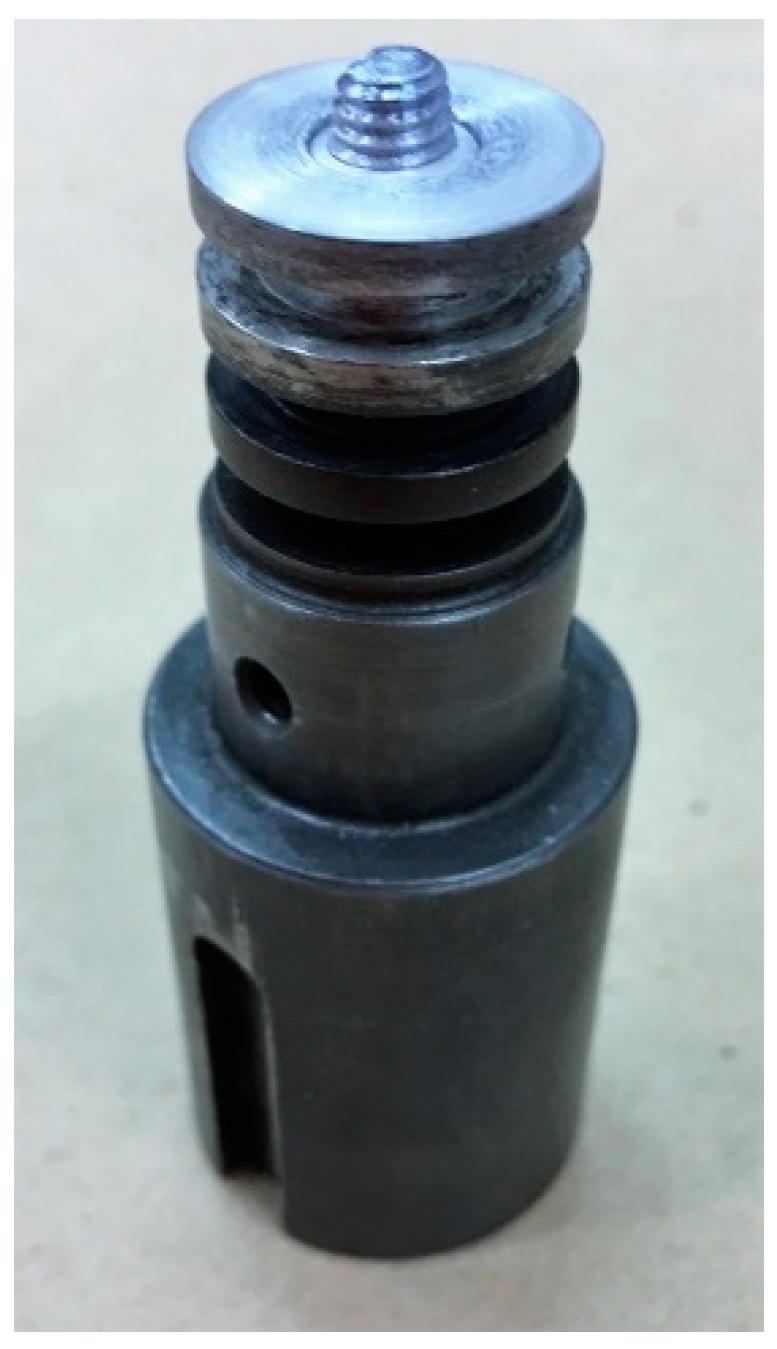
FSW tool used for the welding realization.

FSW was carried out perpendicular to the rolling direction, using a dismountable tool with a flat shoulder in high-speed steel produced by powder metallurgy [28] (Φ = 19 mm) and a tool steel threaded conical pin (Figure 2). The dimensions of the pin were fixed and, in particular, the height was chosen in order to involve both the overlapping sheets.

Welding parameters to make defect-free welds were adopted, based on previous experience [4]. In particular, the tool rotational speed was 630 rpm, the feed speed was 260 mm/min, and a tool tilt angle of 2° was maintained.

### 2.3. Friction Stir Weld-Bonding (FSWB)

**Figure 3:**
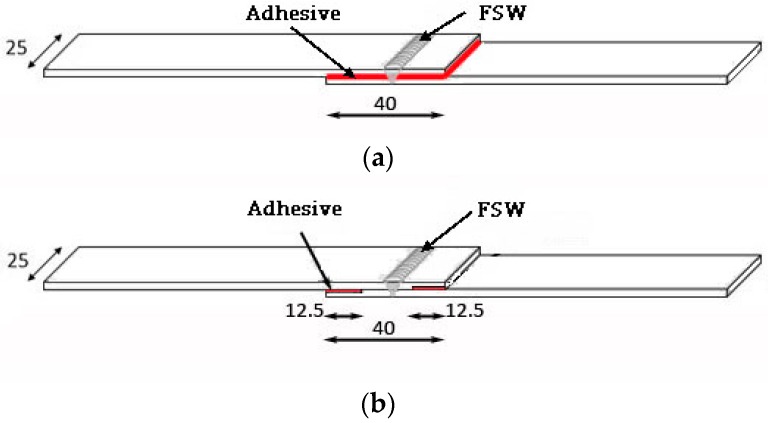
Schematic illustrations of the joints (values in mm), made using two methods: (**a**) Weld-through and (**b**) flow-in.

The application of the adhesive was performed using two methods, suggested by literature on weld-bonding, called the weld-through method (FSWB-WT), in which the adhesive is applied and then the joints are welded through, and the flow-in method (FSWB-FI), in which the welded joint is generated first and then an adhesive is flowed into the area between the sheets by capillarity action [1]. In the first case, the adhesive was applied along the overlapped area, the welding was carried out (Figure 3a), and the crosslinking of the adhesive was achieved at room temperature in one week.

To guarantee the necessary gap for the adhesive application it was necessary to mill the sheets, removing 0.5 mm of material from both plates. In addition, they were prepared using the same sandblasting procedure adopted for AB joints. For FSWB-WT joints, preliminary tests without milling were carried out with unpromising results. In fact, the adhesive was completely expelled during the welding phase due to the pressure acting on the sheets and it also created a thickness that did not allow the correct coupling of the sheets.

Adhesive use in the weld-bonded joints with the weld-through method does not present particular difficulties, since the application of the adhesive is prior to welding. On the contrary, in weld-bonded joints with the flow-in method (Figure 3b), once the welding is done, even after milling the plates, due to their deformation during the welding process, the space available in some areas decreases so much that the introduction of the adhesive is extremely complex. For this reason, application of the adhesive was carried out through a very small nozzle, positioned on the tip of the standard one.

Once this operation had been carried out on both the zones, i.e., above and below the welding bead, the plate of the weld-bonded flow-in joints was cured as the weld-through joints were in order to obtain the correct crosslinking of the adhesive. 

### 2.4. Quasi-Static and Dynamic Tests

To characterize the welded, adhesive-bonded, and weld-bonded joints both for static and dynamic stresses, a hydraulic Instron 8802 (Instron, Buckinghamshire, UK) was used. In particular, tensile tests were carried out according to ASTM D 1002 [29], at a test speed of 1.3 mm/min.

The fatigue tests, carried out according to the ISO 9664 standard [30], were performed with a cyclic load. As a forcing, a sinusoidal wave was used at a frequency of 5 Hz. The variable stresses were kept only tensile in order to have no compression, avoiding the phenomenon of buckling. S–N curves were plotted to have a fairly precise mechanical characterization. It was decided to perform three different load levels, i.e., three different levels of sinusoid amplitude.

## 3. Results and Discussion

### 3.1. Macrographic Analysis

In order to validate the weld-bonding process, the first step was to carry out a macrographic investigation of the joints, which could highlight the distribution of the adhesive in the two methods used and the presence of possible defects. 

**Figure 4:**
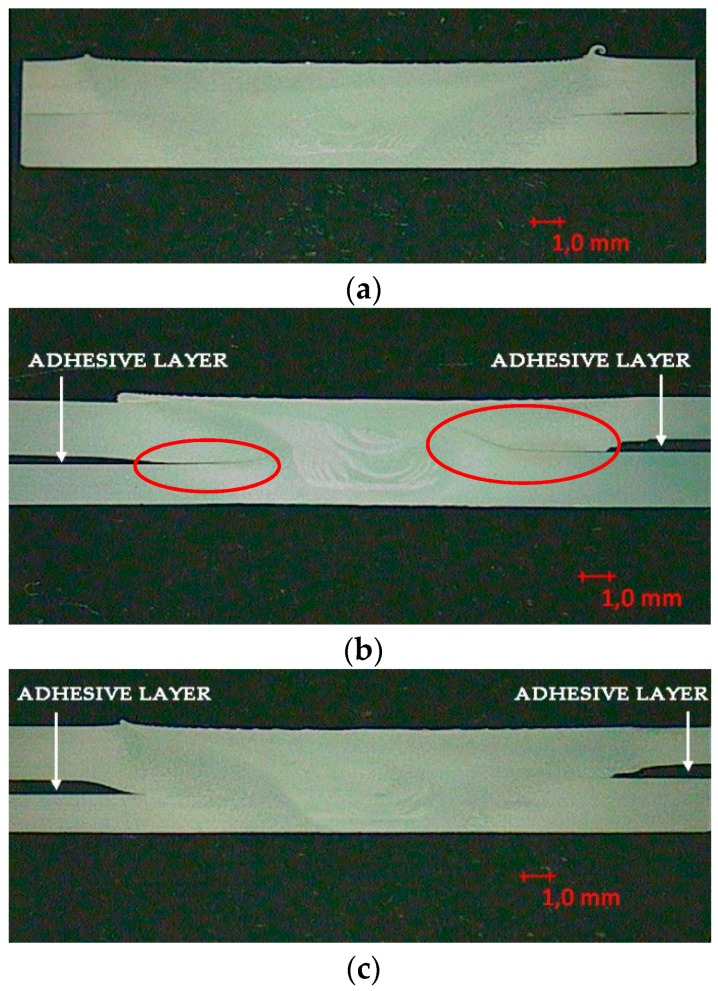
Macrographic inspection of: (**a**) FSW joint; (**b**) weld-through method (FSWB-WT) joint; (**c**) flow-in method (FSWB-FI) joint.

At a first observation, it could be noted that in all cases, the mixing was good, as evidenced by the presence of the classic onion rings. Moreover, the macrographic analysis showed a substantial good synergy between the FSW welding and the adhesive when using the flow-in technique, because in both cases, the joints were found to be free from defects (Figure 4a,c). Furthermore, the adhesive layer, inserted after welding, made it possible to fill the gap and create an obstacle to the propagation of the notch present in all overlapped joints.

On the contrary, the FSWB joint, which was created using the weld-through method (Figure 4b), presented a hook defect, filled with adhesive, created by vertical movement of the material during the FSW process. This is in total agreement with [6], in which many of the hybrid joints made presented a hook defect, which also compromised the mechanical characteristics of the weld-bonded joints, subjected to lap-shear tests.

### 3.2. Single Lap-Shear Tests

**Figure 5:**
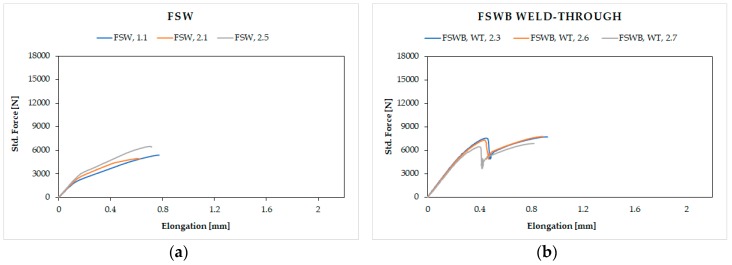

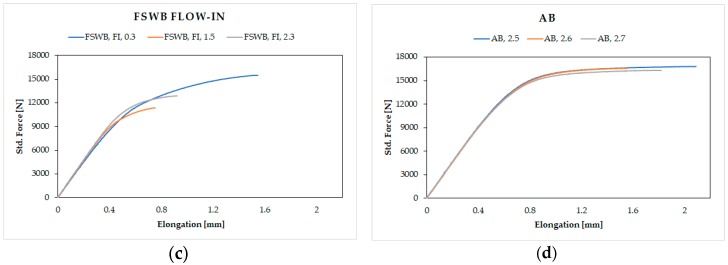
Lap-shear test results for: (**a**) FSW joints; (**b**) FSWB-WT joints; (**c**) FSWB-FI joints; (**d**) adhesive bonding (AB) joints.

Lap-shear strength of the joints was assessed through monotonic testing, using the same test condition for all kinds of joints. Figure 5 reports all the load displacement curves obtained for the different configurations.

Low force values and trend of the tests for the FSW joints (Figure 5a) were expected, due to the intrinsic geometry of the joint that creates a hook, which is a natural fracture trigger. In fact, fractures occur in the heat affected zone (HAZ) for all the samples tested, being the area where the aluminum alloy has undergone a decay of mechanical characteristics, due to the heating induced by the welding process. This is perfectly consistent with what is reported in [6,7,8]. 

The combination of the two technologies in FSWB joints gives different results: The trend of the tensile tests for the joints in which the adhesive was applied before welding over the entire surface (weld-through technique) shows a peak at the failure of the bonding. 

**Figure 6:**
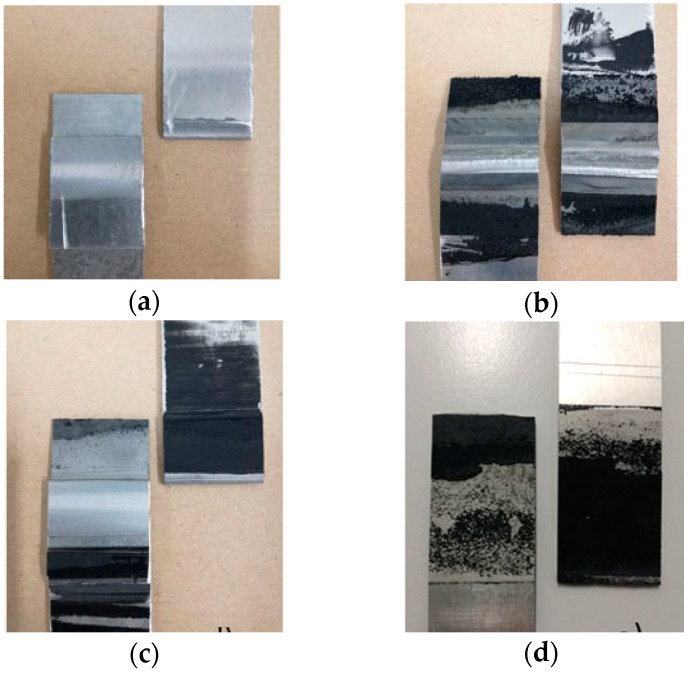
Fracture surfaces of: (**a**) FSW joints; (**b**) FSWB-WT joints; (**c**) FSWB-FI joints; (**d**) AB joints.

In this case, bonding reaches low values of force before fracture. This behavior is mainly due to two phenomena which both contribute to compromising the mechanical response. The first one was highlighted by the macrographic analysis, in which an evident hook defect is present inside the joints and strongly reduces the resistant section; the second is the chemical deterioration suffered by the adhesive during welding. In fact, as Figure 6b shows, the heat generated during welding realization affected the adhesive, which degraded the part between the plates directly under the tool shoulder. Furthermore, also in Figure 6b, an evident lack of mixing caused by the presence of the adhesive, which acts as an insulant and opposes the passage of heat from the upper sheet to the lower one during welding, could be detected. A similar behavior was also observed by Braga et al. [10]. So, this case represents an unfavorable condition from both techniques’ points of view, because simultaneous adhesive damage and incorrect welding between the parts occurred. On the contrary, for the flow-in samples, the maximum peak relative to the yielding of the adhesive revealed by the FSWB weld-through joints is missing. In fact, they show a very uniform curve trend as if the joints were made with a single joining technique, a symptom of excellent coherence between the FSW and the adhesive used. Not only the continuous pattern, but also higher mechanical resistance values were recorded, since the adhesive in this case does not undergo any kind of alteration. It could be concluded that, all FS weld-bonded joints have values of mechanical strength higher than that only welded. Commenting briefly on fracture surfaces in this case (Figure 6c), it could be assessed that the application of the adhesive after welding makes it possible to obtain a well-performing joint with no obvious defects.

As expected, adhesive-bonded joints have higher resistance values because the entire overlap is covered by the adhesive, and the effect of stress concentration is virtually eliminated. 

### 3.3. Fatigue Tests

**Figure 7:**
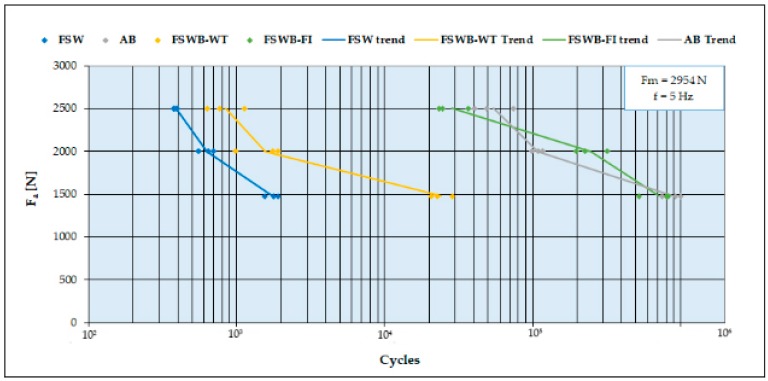
Fatigue resistance comparison between FSW, FSWB (weld-through and flow-in methods) and AB joints.

To investigate dynamic behavior, the same test conditions were adopted for all the joining configurations. On the basis of static tests results, the loads to be applied during the fatigue tests on all types of joints were selected. A mean force F_m_ = 2954 N and three different stress amplitudes F_a_ 2500 N, 2000 N, and 1477 N were adopted. For each of the F_a_ levels, three samples were tested. Figure 7 shows the comparison of fatigue test results on FSW and FS weld-bonded joints, obtained both with weld-through and flow-in methods, as well as AB joints.

The S-N curves in Figure 7 confirm the results of the lap-shear tests. A similar behavior between FSWB-WT and FSW joints could be observed, even if a slight increase of the failure cycles was reported, especially at lower loads. The main explanation of this phenomenon is to be found in the morphology of the overlapped FSW joints, which are characterized by the presence of a notch at the interface between the two plates. This notch has a critical effect, because its role is fundamental in triggering the crack that will break the joint. On the other hand, in the FSWB-WT joints, the adhesive was severely damaged by the thermal cycle, minimizing the generation of intermolecular bonds between resin and substrate during the adhesive cross-linking phase, and therefore failed to significantly contribute to stopping crack propagation, having assumed a very brittle behavior. Furthermore, the adhesive causes a bad mixing of the plasticized material during the welding process. 

**Figure 8:**
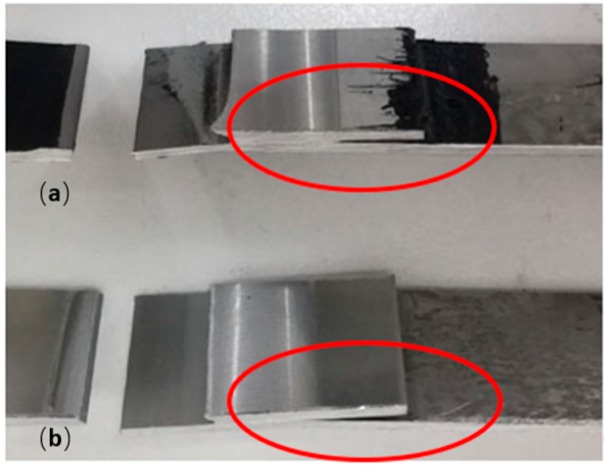
Example of FSWB-FI sample (**a**) and FSW sample (**b**) failures.

Otherwise, the FSWB-FI technique showed a significant improvement in fatigue strength, compared to FSW joints. The considerable difference in fatigue resistance between the two weld-bonding techniques is due to the different execution of the two joining processes. Indeed, in the FSWB-FI joints, the adhesive could synergistically work with the welded joints, opposing cracking at the notch. It should also be noted that the deformation of the plates in the two cases is very different. In the FSW joint, plastic deformation is much more pronounced than in FSWB-FI joints (Figure 8). This causes an additional problem; in fact, the deformation causes a misalignment of the action of the force during the tests, with the consequent creation of a moment that transforms the pure shear stress into a combination of shear and peel. The peel stress greatly reduces the mechanical strength of overlapping joints and causes their breaking for a very low number of cycles. In the case of the FSWB-FI joints, the presence of the adhesive strongly limits the peel stress.

**Figure 9:**
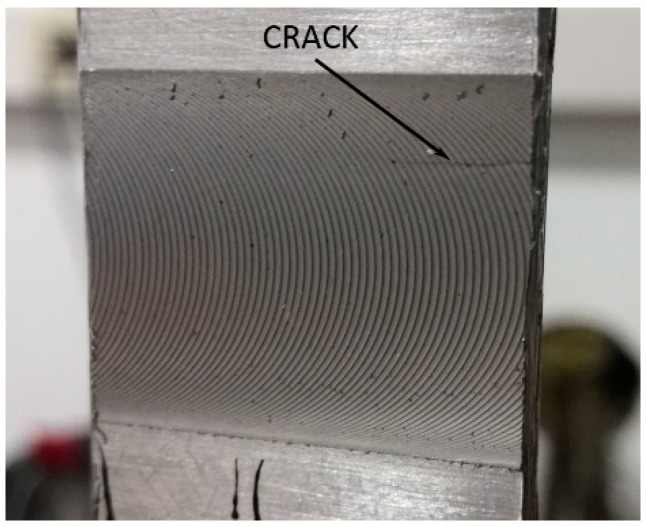
Crack propagation during FSWB-FI sample test.

Finally, an interesting phenomenon that confirms the positive contribution of the presence of the adhesive between the plates, concerns crack propagation during the test. In the FSW joints, once the crack is primed, it progresses very quickly and within a few cycles, a break is reached, while in the FSWB-FI joints, the phenomenon of crack propagation is very slow and it lasts a higher number of cycles, thanks to the damping action of the adhesive. Figure 9 shows the crack during its progress.

## 4. Conclusions

This paper shows the comparison between the static and dynamic stress behavior of AA6082 overlapping joints made with different techniques: Friction stir welding, friction stir weld-bonding, and adhesive bonding.

In particular, the experimental study and analysis of the results allowed us to highlight no problems or disadvantages in the use of the hybrid technique that combines FSW and adhesive bonding. Starting from the realization of the hybrid joints, it emerged that the application of the adhesive before welding is certainly very fast and simple, but it is problematic from the point of view of joint quality, since the heat wave generated during welding is very damaging for the adhesive, which does not withstand such thermal loads. On the contrary, the application of the adhesive after welding makes the machining of the parts to be bonded necessary, which guarantees the presence of the gap necessary to house the adhesive. From a productivity point of view, this last solution is longer, but it is certainly the technique that permits the best combination of the potential of the two technologies. 

The presence of the adhesive in the hybrid joints allows one to compensate for the presence of the structural notch typical of the overlapping FSW, improving both static and dynamic behavior. 

## Figures and Tables

**Table 1:** Chemical composition and mechanical properties of AA6082T6 sheets.

Si	Mg	Mn	Cu	Fe	Cr	Zn	Ti	Other	Al
0.7–1.3	0.6–1.2	0.4–1.0	0.1	0.5	0.25	0.2	0.1	0.15	Bal.

**Table 2:** Chemical composition and mechanical properties of AA6082T6 sheets.

Tensile Ultimate Strength [MPa]	Tensile Yield Strength [MPa]	Elongation at Break [%]	Young’s Modulus [GPa]
290–310	250–260	10	70

**Table 3:** Adhesive data.

Property	Component	
Chemical	Base	Epoxy resin
	Accelerator	Amine modified
Color	Base	Black
	Accelerator	White
Consistency	Base	No sag paste
	Accelerator	No sag paste
Mix ratio	2 part Base: 1 part Accelerator	
